# Supplementary material for: PAUSE: principled feature attribution for unsupervised gene expression analysis
Source: Genome Biol. 2023 Apr 19;24:81. doi: 10.1186/s13059-023-02901-4 (PMC10114348; doi:10.1186/s13059-023-02901-4)
Supplement: Supplementary file 1 — Additional file 1. Figures S1-S12. [file 13059_2023_2901_MOESM1_ESM.pdf]

Additional File 1: Supplementary Figures

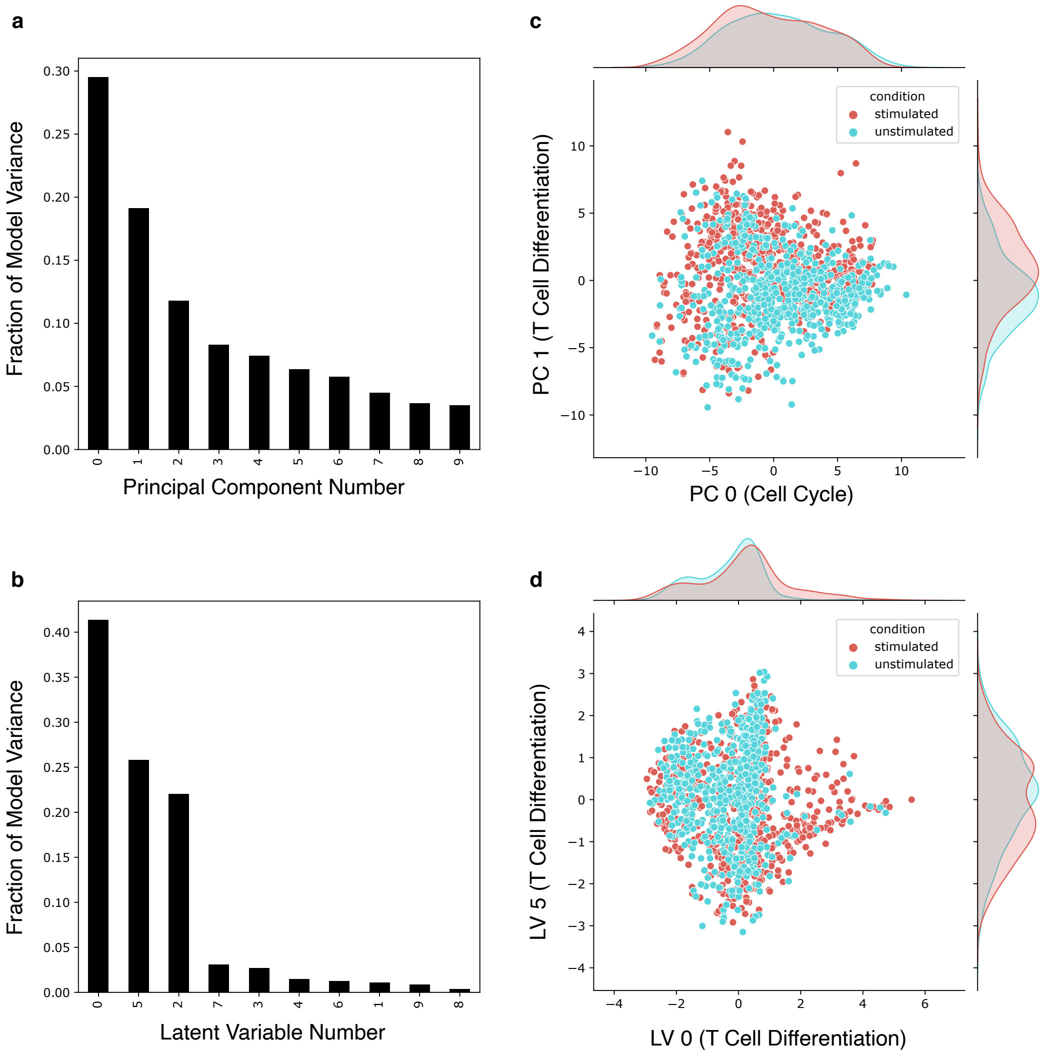

Fig. S1 I **PAUSE Loss Attribution enables analogous workflow to classical models for standard VAEs**. **a**, The fraction of model variance explained by each of the top ten principal components of the Jurkat cell scRNA-seq dataset, calculated using the “explained_variance_” method in the scikit-learn implementation of PCA. Each fraction is equal to the corresponding eigenvalue of the covariance matrix of the original data, divided by the sum of all of the eigenvalues. **b**, The fraction of model variance explained by each of the ten latent dimensions learned by a variational autoencoder trained on the Jurkat cell scRNA-seq dataset, calculated using the global PAUSE loss attribution (averaged across all samples in the dataset), divided by the sum of all of the global loss attributions. **c**, A visualization of the Jurkat cell dataset embedded by the top two principal components. The biological annotations for each of the axes are determined by examining the biological processes enriched in the top magnitude gene loadings for these components, calculated using the enrichments tool on the StringDB web server. **d**, A visualization of the Jurkat cell dataset embedded by the two most important latent dimensions learned by the VAE. The biological annotations for each of the axes are determined by examining the biological processes enriched in the top 100 average magnitude gene attributions for these dimensions, calculated using the enrichments tool on the StringDB web server. A list of all enriched pathways for the principal components and latent variables in **c** and **d** can be found in Additional File 3: Table S2.

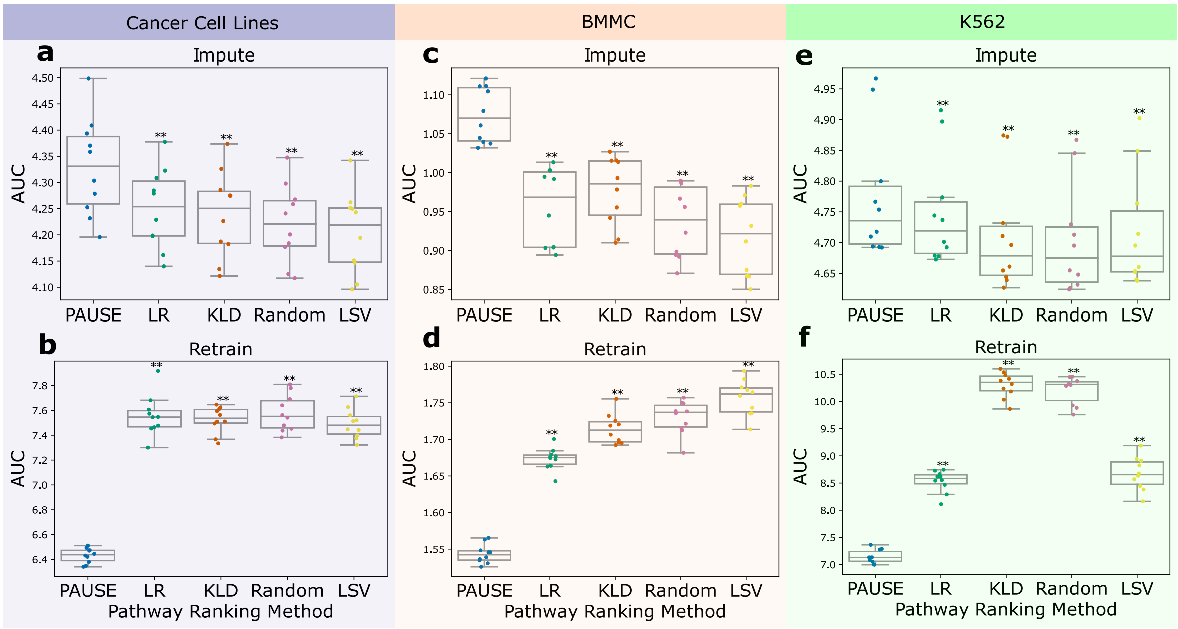

Fig. S2 | **Impute and retrain benchmarks on additional single cell RNA-seq datasets show that PAUSE consistently outperforms other attributions methods**. As in Fig. 2, we apply our "impute" and "retrain" benchmarks to three additional single-cell datasets to verify that PAUSE attributions are better able to capture major sources of transcriptomic variation in a dataset compared to other methods. This datasets contain cancer cells (**a**, **b**), bone marrow mononuclear cells (**c**, **d**), and K562 cells (**e**, **f**). The other methods shown here are logistic regression score (LR), Kullback–Leibler divergence (KLD), random ranking, and latent space variance (LSV). On the impute benchmark, better methods increase reconstruction error faster, leading to a larger AUC. On the retrain benchmark, better attribution methods decrease reconstruction error faster, leader to a smaller AUC. In these analyses, we find that PAUSE consistently outperforms the other methods (two-sided Wilcoxon signed rank test, Bonferroni corrected p=7.81×10^{-3}, statistic=0.0).

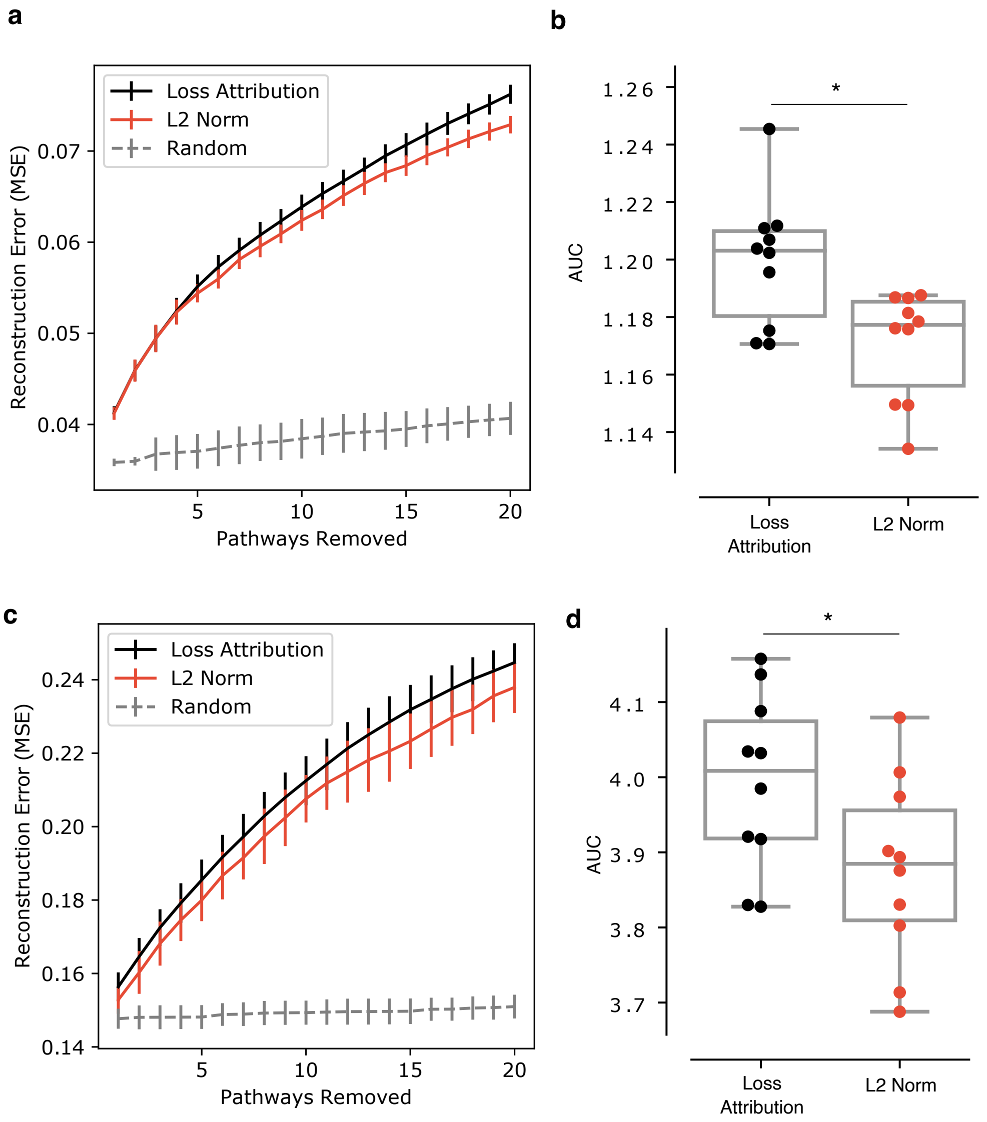

Fig. S3 | **Impute benchmark for VAE architecture with a linear decoder**. **a**, As top pathways ranked by our PAUSE Loss Attribution, ranked by the L2 norm of the weights in the decoder, or ranked randomly are removed by mean imputation, the reconstruction error (MSE) of a VAE with a linear decoder trained on the PBMC dataset increases. Error bars indicate the standard deviation when the benchmark is re-run over 10 independent randomizations of the dataset into train/val/test splits. **b**, Summary of the area under the imputation curves in **a** for loss attribution and L2 norm. The boxes mark the quartiles (25th, 50th, and 75th percentiles) of the distribution, while the whiskers extend to show the minimum and maximum of the distribution (excluding outliers). Loss attribution identifies important pathways significantly better than the L2 norm (Wilcoxon rank-sums test statistic = 2.19, p = 0.028). **c**-**d**, These subpanels show the same information as **a**-**b**, where this time the benchmark (again using a VAE with a linear decoder) is run on the dataset of stimulated Jurkat cells. Again, PAUSE identifies important pathways significantly better than the L2 norm (Wilcoxon rank-sums test statistic = 1.97, p = 0.049).

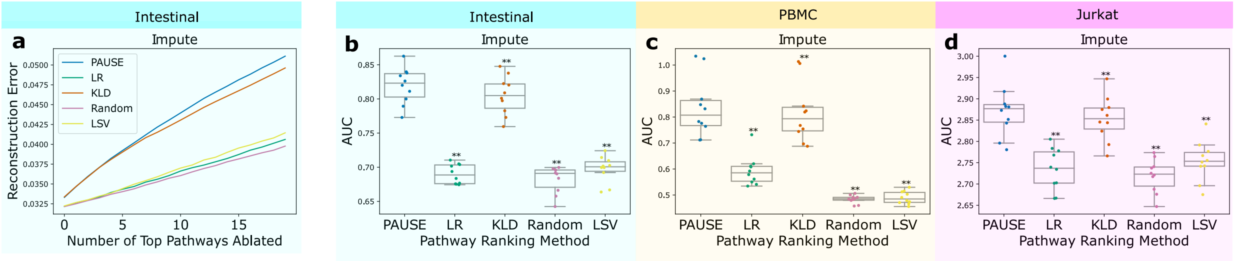

Fig. S4 l **Impute benchmark for standard VAE architecture**. The results of our impute (**a**) benchmark on a standard VAE show that PAUSE attributions still outperform other attribution methods on single cell RNA-seq datasets comprised of intestinal (**b**), peripheral blood monocytes (**c**), and Jurkat cells (**d**). This benchmark was performed as described in main text Fig. 3, however with a standard VAE instead of a pmVAE model. In this standard VAE model, dense connections exist from every input node to every node in the hidden layer. The number of nodes in this model's layers is the same as in the pmVAE benchmark experiments, as described in the Methods. We find that PAUSE attributions outperform other attribution methods (two-sided Wilcoxon signed rank test, Bonferroni corrected p=7.81×10^{-3}, statistic=0.0). We also note the improved performance of KLD as an attribution method in this context, when the VAE is optimized only to minimize divergence from an isotropic Gaussian prior and reconstruct the input, compared to the pmVAE model that is trained with additional biological latent space constraints (and a decreased penalty for the KL divergence term).

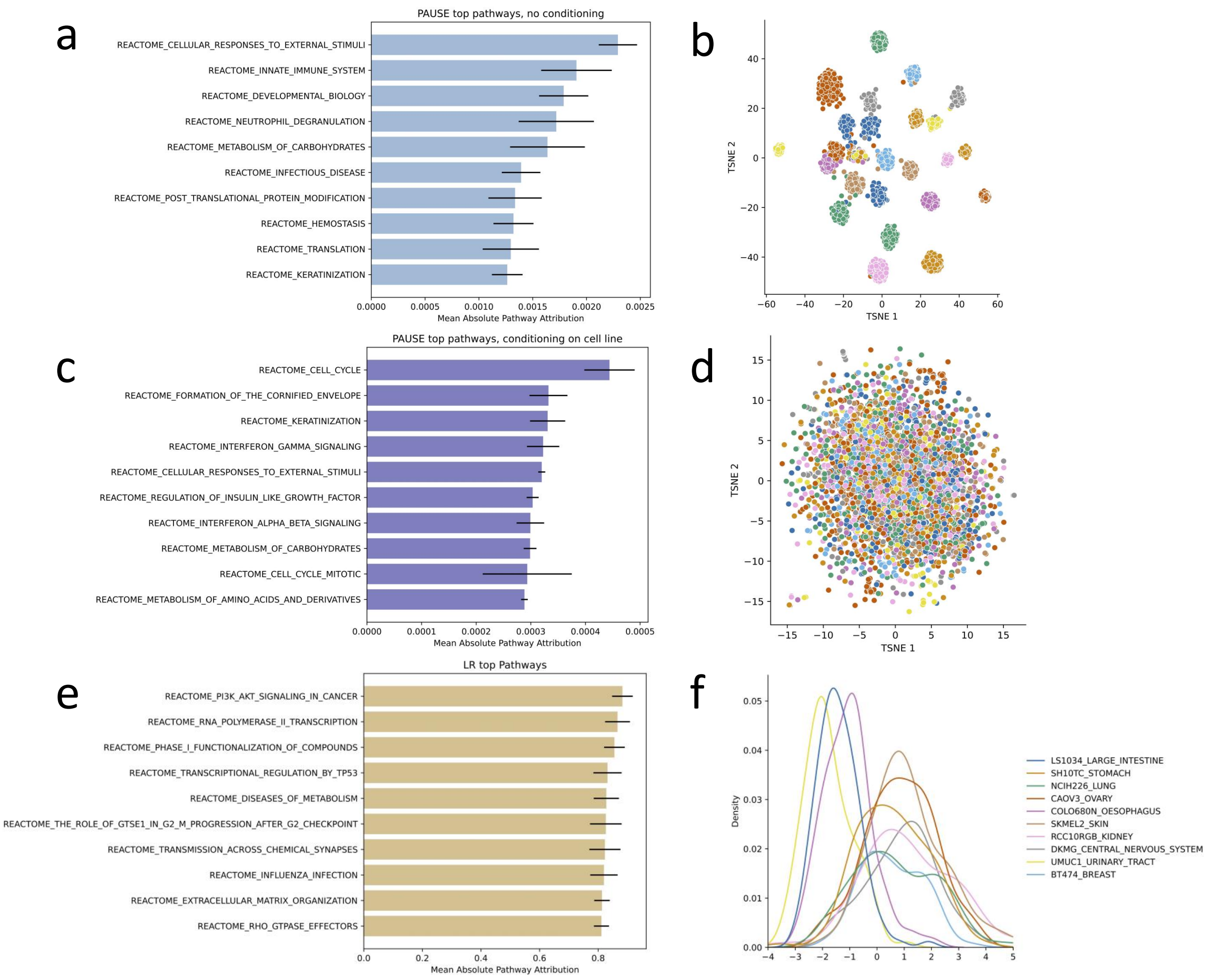

Fig. S5 | **PAUSE Loss Attribution allows for analysis of biological signal beyond labels provided**. **a**, The top pathways identified by PAUSE from a pmVAE trained on the cancer cell lines dataset from McFarland et al. Many of these pathways, like Innate Immune System or Developmental Biology are not specific to the activity of TP53, but rather appear to relate more to differences between the tissues of origin of the 24 cell lines in the dataset. **b**, A t-SNE plot of samples embedded in the pmVAE latent space, colored by cell lines, shows that the latent space separates by cell line. **c**, When we use a *cpmVAE* to take explicitly account for cell line and thus allow the model to learn the perturbation of interest in the latent space, the PAUSE top pathway becomes cell cycle, which fits with our understanding of how p53 acts to induce apoptosis. **d**, A t-SNE plot of samples embedded in the *cpmVAE* latent space, colored by cell lines, shows that the latent space is of this model is not separated by cell line. **f**, A closer analysis of a PAUSE top pathway, Innate Immune System, in the non conditional pmVAE model shows that embedding values for this pathway fall into distinct distributions based on cell lines' tissues of origin, rather than capturing the biological signal given by the TP53 status label. This example shows the utility of PAUSE providing a fully unsupervised method that is capable of selecting pathways most relevant to transcriptomic variation without using labels. In this case, differences between cell lines represent an additional source of biological signal beyond the difference in TP53 status. Using labels of only one specific biological signal to rank top pathways has the potential to miss other important sources of variation, which is why unsupervised attributions have an important use, even when labels do exist.

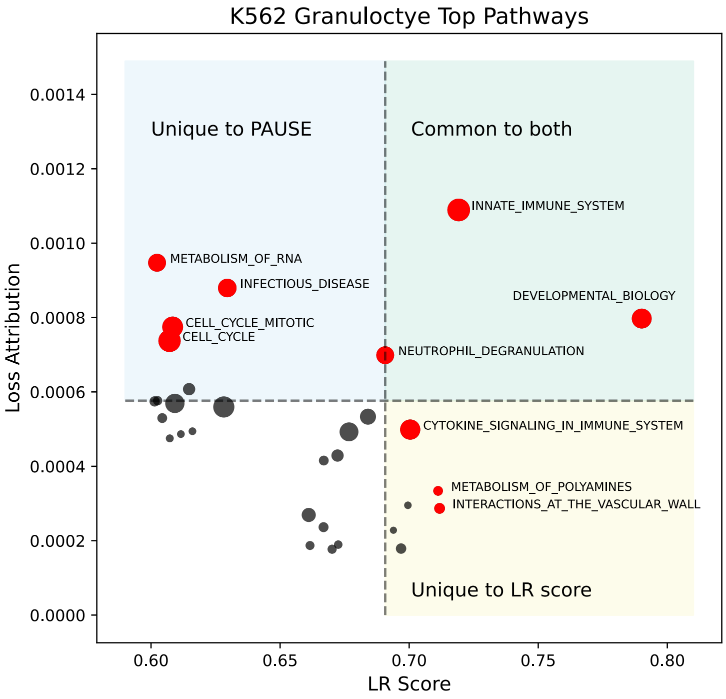

Fig. S6 I **Analysis of K562 dataset shows additional gain from unsupervised analysis**. Here, we show top pathways identified in the K562 dataset by unsupervised (PAUSE attributions) and supervised (logistic regression) techniques. In this dataset, the authors use Perturb-seq (single-cell RNA-sequencing pooled CRISPR screens) to perturb single genes and pairs of genes in K562 cells and measure transcriptional response. For this analysis, we apply a pmVAE model to a dataset consisting of unperturbed cells (serving as a control) as well as cells labeled by the authors as belonging to the “granulocyte/apoptosis” cluster of gene perturbations due to their expression of granulocyte and apoptosis markers. The annotation of either "control" or "granulocyte/apoptosis" serves the label for the supervised analysis. We find that some pathways identified as important by both supervised and unsupervised techniques, like Innate Immune System and Neutrophil Degranulation, capture the expected variation between control cells and those perturbed to express granulocyte markers. In addition, other top PAUSE pathways that are not top supervised pathways, like Cell Cycle and Cell Cycle Mitotic reflect the mechanism of the perturbation and the noted cell deaths caused by these perturbations. Many perturbed genes are cell cycle regulators, and the original authors show deviation of cell cycle as a clear effect of the perturbations. Supervised attributions also pick up on other pathways relevant to the “granulocyte” label, like Cytokine Signaling, but do not include the transcriptomic variation due to cell cycle changes. Again, this example illustrates that supervised attributions are useful to the extent that we have labels defining the specific source of transcriptome variation we are interested in. To further explore other sources of variation, unsupervised attributions are of additional use.

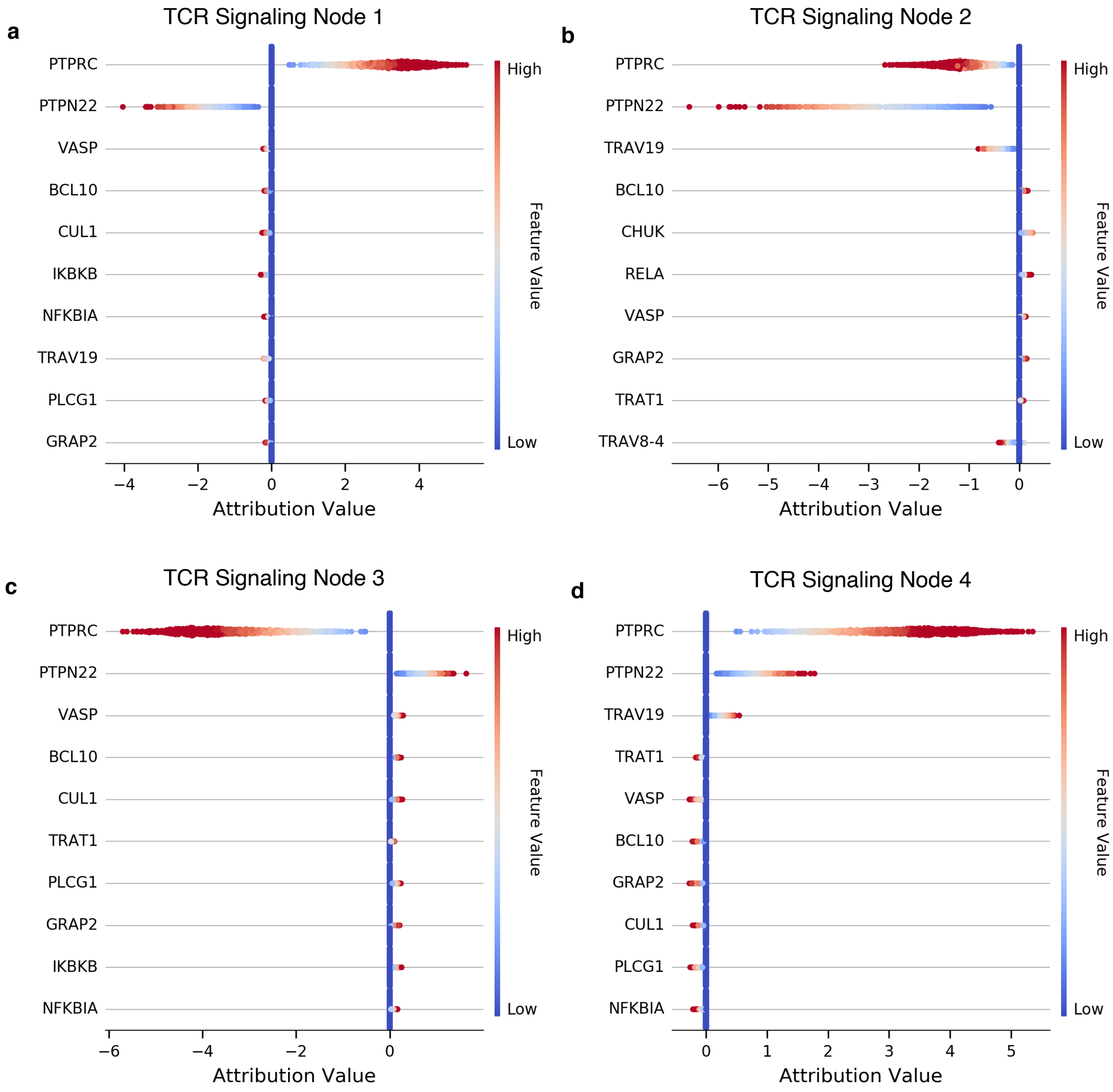

Fig. S7 I **Summary plot of top genes for TCR Signaling Module**. The four summary plots show swarmplots of the distributions of gene attributions for the four nodes in the TCR Signaling module for a pmVAE model trained on the single cell dataset of CD3/CD28-stimulated Jurkat cells. Each point is a single sample, the x-axis of each plot shows the attribution for that gene for that sample, and the color encoding shows whether the expression level of that gene is high or low in the sample.

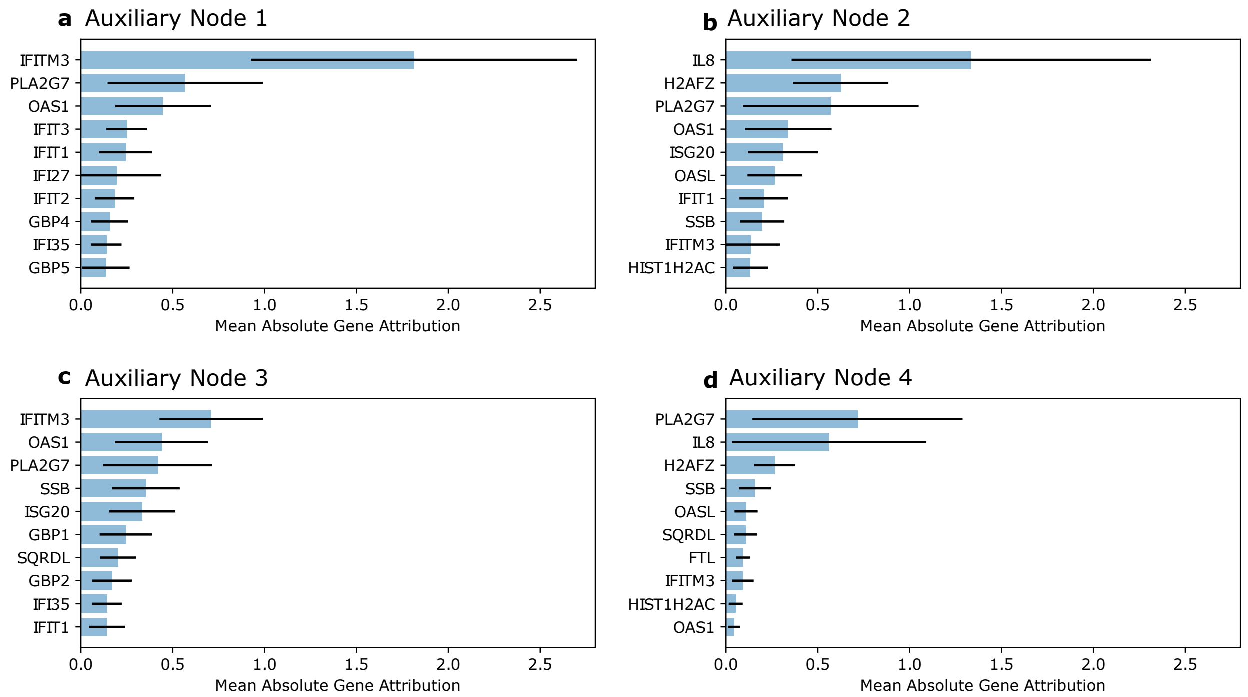

Fig. S8 | **Inteferon-related genes are identified as top genes in auxiliary nodes when Inteferon-related pathways are not included as pathway modules**. In this experiment, we trained the network without main Interferon-related pathways ('REACTOME ANTIVIRAL MECHANISM BY IFN STIMULATED GENES', 'REACTOME DDX58 IFIH1 MEDIATED INDUCTION OF INTERFERON ALPHA BETA', 'REACTOME INTERFERON GAMMA SIGNALING', 'REACTOME INTERFERON ALPHA BETA SIGNALING','REACTOME INTERFERON SIGNALING') for the PBMC single cell dataset. We then examined the densely connected auxiliary pathway, which is comprised of 4 pathway nodes. Shown here are the top 10 most important genes by mean absolute gene attribution value for each of the four nodes, with error bars representing standard deviation.

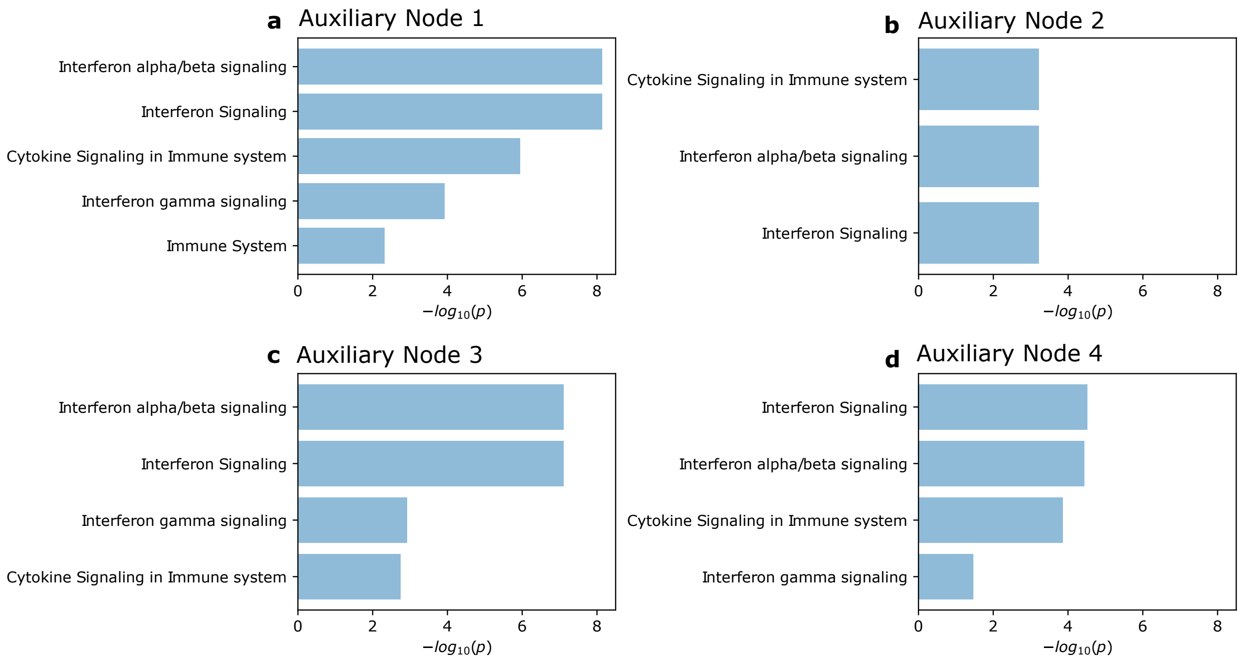

Fig. S9 I **Genes from the pathway 'Regulation of PLK1 Activity at G2/M Transition' are not identified as top genes in auxiliary nodes when this pathway is not included as a pathway module**. For each of the four auxiliary nodes (when the network is trained without Interferon pathway information), we generated gene-level attributions to obtain the top 50 most important genes for each node. We then used the STRINGdb web tool to analyze Reactome pathway enrichments for these top genes for each node compared to all genes in the dataset. In plots **a-d**, we show -$\log_{10}$ p values for pathways with $p<0.05$ when corrected for multiple testing using the Benjamini–Hochberg procedure.

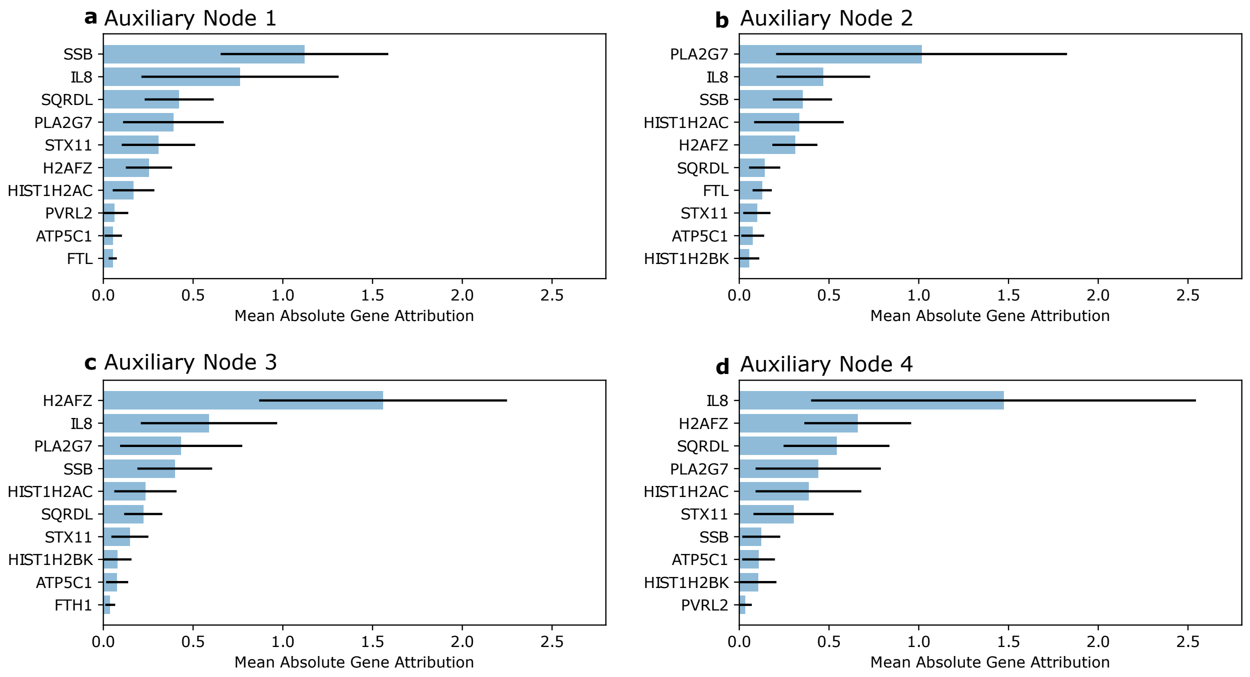

Fig. S10 | **Interferon-related pathway enrichments are found in auxiliary node top genes when Interferon-related pathways are not included as pathway modules**. In this experiment, we trained the network without the pathway 'REACTOME REGULATION OF PK1 ACTIVITY AT G2/M TRANSITION' for the PBMC single cell dataset. We then examined the densely connected auxiliary pathway, which is comprised of 4 pathway nodes. Shown here are the top 10 most important genes by mean absolute gene attribution value for each of the four nodes, with error bars representing standard deviation.

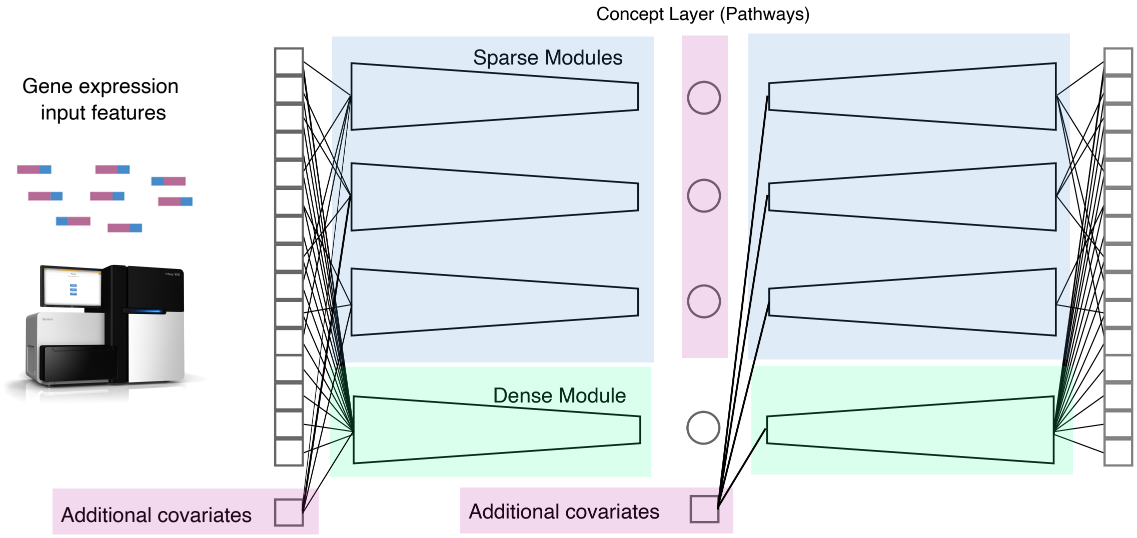

Fig. S11 l **Conditional pathway module VAE allows for the correction of undesired sources of variation**. In order to enable our model to correct for unwanted sources of variation in gene expression data, it was necessary to modify the pmVAE architecture to be able to condition on nuisance variables. The architecture of our conditional pathway module VAE (cpmVAE) is similar to the pmVAE architecture, in that the internal nodes of the network are separated into modules corresponding to each pathway. Within each pathway module the intermediate nodes are all densely connected, and between each module there are no connections between nodes. In the bottleneck layer, while there is only one latent node per module depicted in the feature, there can be any number of latent nodes per module. At the input and output layers, each module is connected to the input genes or the reconstruction genes with sparse connections ensuring that genes are only connected to the pathway modules that they are annotated to belong to. Dense modules are connected to all genes. The modification to allow for variable conditioning is to pass in a vector of additional covariates, which in addition to the genes are provided as an input to every module at both the input layer and again in the bottleneck layer.

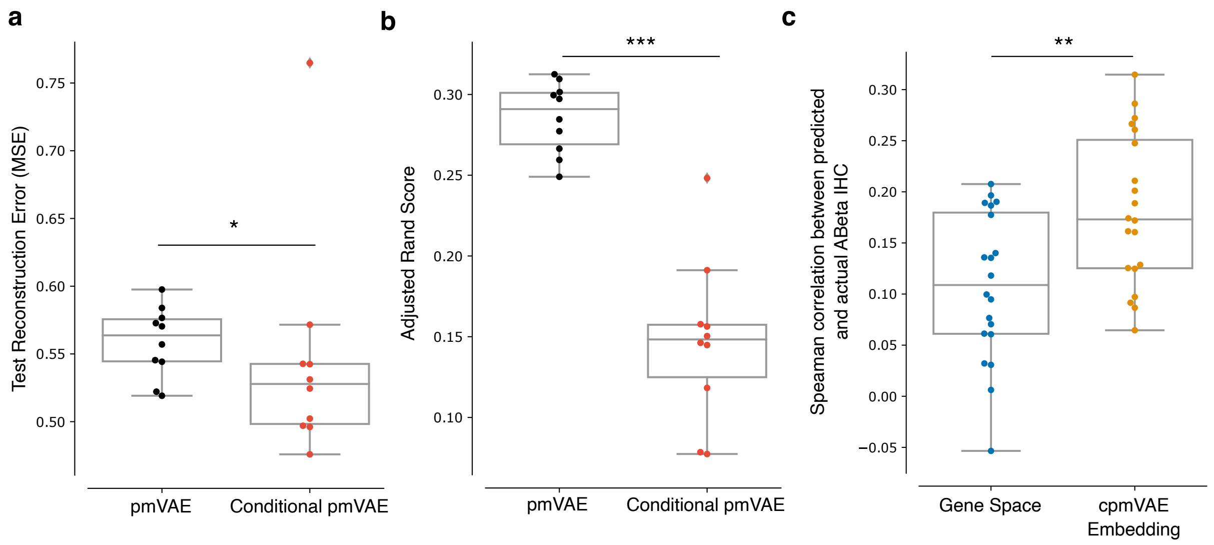

Fig. S12 | **Sanity checks ensure biological validity of cpmVAE model on AD data**. **a**, Comparison of reconstruction error attained by pmVAE model and our conditional pmVAE (cpmVAE) model on the AD dataset. For 10 different random train/test splits, we trained both a pmVAE and a cpmVAE on the training set for 50 epochs, measured the reconstruction on the held out test set for each model, and compared the distribution of test reconstruction errors for the two models. The distribution of test reconstruction errors attained with the cpmVAE model is significantly lower than the distribution of test reconstruction errors attained by the previously proposed pmVAE model (two-sided Wilcoxon rank-sums test statistic = 1.96, p = 0.049). **b**, Comparison of adjusted Rand indices (ARIs) of latent space clusters by dataset source between pmVAE and cpmVAE models. The distribution of ARIs for the pmVAE embeddings were significantly larger than the distribution of ARIs for the cpmVAE embeddings, indicating that the embedding learned by the cpmVAE model has significantly less clustering according to dataset source (two-sided Wilcoxon rank-sums test statistic = 3.78, p = 1.57×10^{-4}). **c**, For 20 random train/test splits of the AD data, we compared the Spearman correlation between predicted and actual Aβ density for linear models trained on the full gene expression measurements to the Spearman correlation between predicted and actual Aβ density for linear models trained only on the cpmVAE pathway embeddings. The cpmVAE embedding features lead to significantly more predictive models of Aβ density than the original gene expression features (two-sided Wilcoxon rank-sums test statistic = 2.81, p = 4.90×10^{-3}). In each plot, the boxes mark the quartiles (25th, 50th, and 75th percentiles) of the distribution, while the whiskers extend to show the minimum and maximum of the distribution (excluding outliers).
